# Digital Mirror Device Application in Reduction of Wave-front Phase Errors

**DOI:** 10.3390/s90402345

**Published:** 2009-03-30

**Authors:** Yaping Zhang, Yan Liu, Shuxue Wang

**Affiliations:** 1 Department of Physics, School of Science, Kunming University of Science and Technology, No. 68, Wenchang Road, 121 Street, Kunming, Yunnan Province. ZIP: 650093, P.R. China; E-mail: yaping.zhang@gmail.com; 2 Air Force Engineering University, Xi’an 710077, P.R. China; E-mail: type407@163.com; 3 Kunming Metallurgy College, Kunming 650033, P.R. China; E-mail: pingzy@126.com

**Keywords:** DMD, PDS, wavefront phase error, optical correction, phase aberrations

## Abstract

In order to correct the image distortion created by the mixing/shear layer, creative and effectual correction methods are necessary. First, a method combining adaptive optics (AO) correction with a digital micro-mirror device (DMD) is presented. Second, performance of an AO system using the Phase Diverse Speckle (PDS) principle is characterized in detail. Through combining the DMD method with PDS, a significant reduction in wavefront phase error is achieved in simulations and experiments. This kind of complex correction principle can be used to recovery the degraded images caused by unforeseen error sources.

## 1. Introduction

The optical window is an important element in high-speed aircraft to receive the target signal. When light-rays pass through a window, refractive index distribution in the window is non-uniform due to the thermo-optic and elastic-optic effects. It is the major factor in the optical quality. Therefore, finding the influencing factor(s) and correcting the distorted wavefront are of great significance to enhance the detection precision of high-speed aircraft [1,2]. Sun has put forward the method of image reconstruction using some reasonable prior information to regularize the atmosphere turbulence degradation model (point spread function). She estimated the PSF values with the ARTUR algorithm [3]. A blind correction method based on the match of feature points was proposed, which is related to the problems of degraded images caused by aero-optics effects. But the local area correction was almost non-uniform [4]. When the adaptive optics wavefront correction method is used, a deformable mirror must correct its own deformation before it could correct other one [5].

In this paper, a complex method is described to correct the wavefront aberration combining DMD based on MOEMS with PDS principle [6]. DMD can be used as the spatial light modulator to construct a digital holographic display system [7],[8]. At the same time, DMD has a wide applicability in optical information processing and structured illumination three-dimensional sensing [9]∼[14]. In the imaging processing field, PDS is a novel method. This approach blends the strengths of speckle-imaging and phase-diversity concepts. Compared with other methods such as pure digital image processing (Wiener filtering de-noising, wavelet de-noise, etc.) and opto-electronic processing methods (such as wavefront detectation in AO, speckle imaging, etc.) PDS has the following advantages: first, the optical hardware is compact. Second, the method is less susceptible to systematic errors introduced by optical hardware. Third, this approach also fits well for extended objects. It can be forecasted that PDS has better prospects in application to reduce aberration and distortion caused by the atmosphere turbulence and miscellaneous random factor [15,16]. Finally, through experiments with DMD and PDS, the modulation transfer function (MTF) and point spread function (PSF) results show that the quality of the restored images was obviously improved.

## 2. Correction Principle and Method

### 2.1 First step: adaptive optical correction using DMD

**Figure 1.**
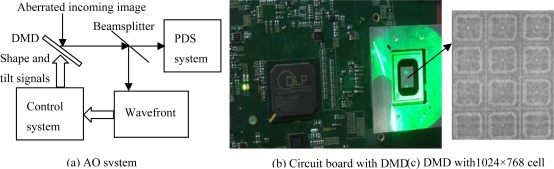
Using DMD to correct distorted wavefront in AO system

**Figure 2.**
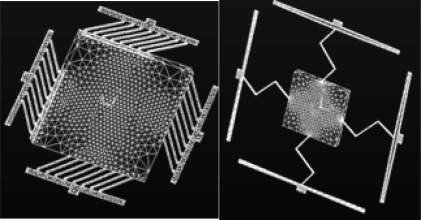
Cell sensor actuator.

The AO system is a closed-loop system, which controls the optical wavefront in real time, (see Figure 1) [17,18]. DMD can provide high resolution images with a wide field of view. It can enhance the system by monitoring actuator in closed loop control and automatically adjusting the output based on the feedback data. The core of a DMD is an array of aluminum mirrors that reflect the light. Such a micro mirror array is composed of thousands of mirrors with an edge length of about 13 μm mounted on small hinges atop a CMOS device. The individually mirrors can be tilted between two positions. This DMD has 1,024×768 cells and be controlled by SLM and DLP. The cell sensor actuator is analyzed and optimized with ANSYS software like in Figure 2. Here, SLM is used to modulate the phase and amplitude of incident light through active control of mirror array. DLP slug controls each actuator of mirror. Both of them can modulate the spatial phase of the wavefront.

From the first correction step, we can get the amplitude and intensity distribution of the incident wavefront. Assumption, the complex amplitude distribution of the pupil plane of the AO system is:

(1) $$E_{0}(\rho ,t)=A(\rho )\text{exp}[(j\frac{2\pi }{\lambda }W(\rho )]$$

Here, *A*(*ρ*) is the distribution function of incident wave amplitude; *W*(*ρ*) is the wavefront aberration function of the incident wavefront. After the AO system, the lights are focused at the image plane, so the amplitude distribution is:

(2) $$E(r)=\frac{\text{exp}[(\rho^{2}+z^{2})/z\lambda }{j\lambda z}\int E_{0}(\rho )\text{exp}[-j\frac{2\pi }{\lambda z}r\cdot \rho ]d\rho$$

and the light intensity distribution is:

(3) $$I(r)=\frac{1}{\lambda^{2}R^{2}}{\left|\int A(\rho )\text{exp}[j\varphi (\rho )]\times \text{exp}[-2\pi jr\cdot \rho /\lambda R]d\rho \right|}^{2}$$

### 2.2 Second step: PDS correction

By analyzing the relation between the Zernike coefficient and the aberration reason, according to the wavefront recovery algorithm, we obtained the relationship of Zernike coefficient and the wave function.

**Figure 3.**
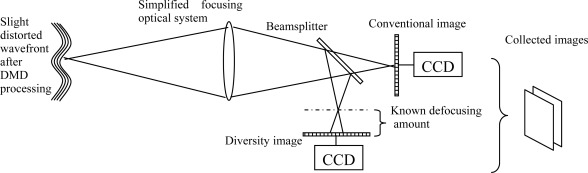
Data-collection scheme for phase diverse speckle imaging.

After the first processing step, the wavefront still is slightly distorted. To further correct the wavefront and reduce the image blurring, a novel method - phase diversity speckle (PDS) technique - is presented. The PDS method is less introducing systematic errors by optical hardware. The principle scheme is seen in Figure 3. The incident light comes from the output result of AO system. For recovery of the original image information, PDS only requires two images. One of them is the conventional focal-plane image, which is degraded by some unknown aberration, and the other is formed by an aberration with a certain known mode, such as at a known defocused position [19,20]. Through establishing the phase aberration function by Zernike polynomials, the adaptive genetic algorithm is adopted to search the global optimum point of the object function, so Zernike coefficients is evaluated [21]. PDS uses each photon to form the image and to detect aberration. It does not calculate the mean value of the image, so the principle can be used in applications to recover degraded images caused by unforeseen sources. From Figure 3 we can see that the optical setup is simple, and it performs well with extended objects, so we do not worry about the dimension of the light source. Through math deduction, the target function can be expressed as:

(4) $$F(\alpha )=\frac{D_{1}(u)S_{1}^{*}(u;\alpha )+D_{2}(u)S_{2}^{*}(u;\alpha )}{{\left|S_{1}^{*}(u;\alpha )\right|}^{2}+{\left|S_{2}^{*}(u;\alpha )\right|}^{2}}$$

where, *D*_1_ is the spectral function of the conventional image; *D*_2_ is the spectral function of the diversity image; $S_{1}^{*}(u;\alpha )$ is the estimated value of the transfer function of the conventional optical path; $S_{2}^{*}(u;\alpha )$ is the estimated value of the transfer function of the defocusing optical path. The evaluation function is defined as:

(5) $$F/F(\alpha )=10\text{lg}\frac{\sum_{M=1}^{M}\sum_{N=1}^{N}{\left[f(m,n)\right]}^{2}}{\sum_{M=1}^{M}\sum_{N=1}^{N}{\left[f\prime (m,n)-f(m,n)\right]}^{2}}\;\;\;\;\;\;\;\;(\text{dB})$$

Here *f*(*m*,*n*) is the original image; *f′*(*m*,*n*) is the corrected image. The value of *F / F*(*α*) represents the distortion degree of the corrected image relative to the original image.

## 3. Experimental Results

A laboratory simulation experiment was completed by the software ANSYS and MATLAB. The influence of the optical window under aero-optical condition was studied. The ray-tracing program crossing the optical window with non-uniform refractive index is programmed. And the wave front chart is drawn. Then combining the DMD and PDS techniques, the objective function is evaluated. The optical correction is studied according to the results of simulation and experiments.

**Figure 4.**
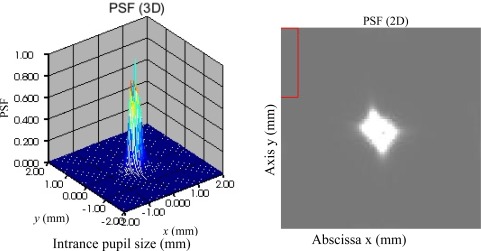
PSF of the distorted wavefront.

**Figure 5.**
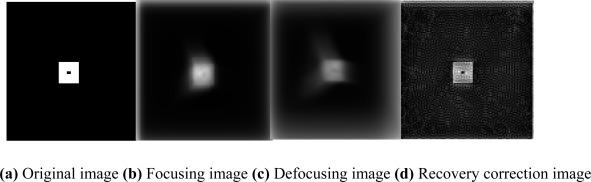
Simulation result of the PDS correction method.

**Figure 6.**
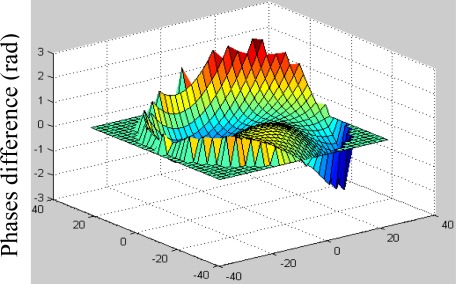
The difference between initial and estimated phases(left).

**Figure 7.**
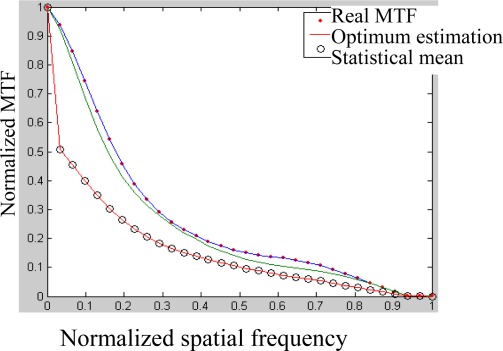
Real, the best estimated and mean MTF in10 simulations (right).

**Figure 8.**
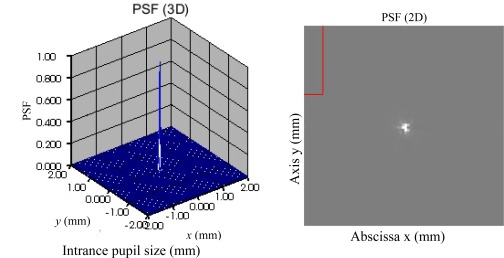
PSF of the corrected wavefront.

For easy to analyze the correction result, the PSF (3-dimension and 2-dimension) of the distorted and corrected wavefronts are given in Figures 4 to 8. Figure 5 is the result of the DMD and PDS correction method. From Figures 4 and 8, we can see the correction result is satisfied.

From the comparison between Figures 5(a) and (b), we can see the recovery correction image is clear-cut relative to the original image.

Figure 6 is the difference between initial and estimated phases. From which we can see the differences or the aberration is slight and the MTF of Figure 7 and PSF of Figure 8 are satisfied.

## 4. Conclusions

This paper presents an overview of research and development progress in MOEMS and PDS for optical correction of aero-optics. The resolution of an incoherent diffraction-limited imaging system is often limited by phase aberrations. Phase aberrations arise from a variety of sources. These unknown phase aberrations can corrupt the wavefront and result in bore-sight and centroid errors for tracking systems, blur and identification problems for imaging systems, and defocus and jitter for directed energy systems, any of which can substantially impact mission effectiveness. The complex method presented is less susceptible to systematic errors introduced by optical hardware, and it also works well for extended objects. Based on the experimental result, significant reduction in wavefront phase error is achieved.
